# Long-term prediction of fish growth under varying ambient temperature using a multiscale dynamic model

**DOI:** 10.1186/1752-0509-3-107

**Published:** 2009-11-10

**Authors:** Nadav S Bar, Nicole Radde

**Affiliations:** 1 Department of Chemical Engineering, Norwegian University of Science and Technology, NO-7491, Trondheim, Norway; 2 Institute for Systems Theory and Automatic Control, University of Stuttgart, 70550 Stuttgart, Germany

## Abstract

**Background:**

Feed composition has a large impact on the growth of animals, particularly marine fish. We have developed a quantitative dynamic model that can predict the growth and body composition of marine fish for a given feed composition over a timespan of several months. The model takes into consideration the effects of environmental factors, particularly temperature, on growth, and it incorporates detailed kinetics describing the main metabolic processes (protein, lipid, and central metabolism) known to play major roles in growth and body composition.

**Results:**

For validation, we compared our model's predictions with the results of several experimental studies. We showed that the model gives reliable predictions of growth, nutrient utilization (including amino acid retention), and body composition over a timespan of several months, longer than most of the previously developed predictive models.

**Conclusion:**

We demonstrate that, despite the difficulties involved, multiscale models in biology can yield reasonable and useful results. The model predictions are reliable over several timescales and in the presence of strong temperature fluctuations, which are crucial factors for modeling marine organism growth. The model provides important improvements over existing models.

## Background

Efficient and accurate prediction of whole body growth is very important in animal husbandry, particularly in marine aquaculture, a rapidly developing and important source of food. Feed development is largely dependent on accurate growth prediction, and it has significant economical and environmental consequences. However, the growth of living organisms is a complex process, affected by both external and biotic factors. Thus, organism growth is extremely difficult to predict, particularly over long periods of time. Mathematical models of varying complexity often serve as tools for the prediction and simulation of animal growth [1,2], although usually with a short prediction horizon of several days (see, for instance, [3,4]). Earlier attempts to describe whole body growth were mainly conducted for the design of feed and were mostly based on empirical functions (see, for instance, [5-7]). These models were largely based on data sets derived from multiple experiments and were valid only under specific conditions. Thus, they could only be applied with confidence within a relatively small range of conditions. For example, growth in pigs, cattle, and chickens has been addressed using several dynamic models developed in recent years [8,9]. These simple models effectively predicted growth and body composition under a narrow range of environmental conditions. A few mechanistic models were also developed to simulate the growth of fish [3,10,11]. These models did not attempt to describe protein metabolism or the regulatory mechanisms of the animal, and they could not predict features such as amino acid (AA) retention, an important nutritional and economic property. Moreover, the models did not take into account the dependence of protein metabolism on environmental factors, although these have been shown to have a strong impact on marine organisms. In particular, fluctuations in temperature strongly influence marine organisms. Most marine organisms are ectothermal, and their growth response is highly sensitive to variations in water temperature [12]. Temperature affects protein synthesis in a linear manner [13-15]; however, despite a large number of studies, temperature's effect on protein degradation is still unknown, probably due to the difficulty of measuring *in vivo *rates of protein degradation [16]. This temperature-degradation rate relationship is critical to any study of the growth of aquatic life forms.

Complex regulatory networks affect most inter- and intracellular processes, including cell growth [17]. Nutrients from the feed intake are mobilized and utilized *de facto *on many levels of regulation, which also respond to external stimuli. Regardless of their framework, earlier predictive models incorporated neither cell regulation nor environmental effects, which are naturally not multiscaled. Conversely, structured mathematical models that do incorporate regulations recurrently have elucidated the link between the processes that affect homeostasis and growth [18]. Such a framework also has practical applications due to the potential for the accurate prediction of growth and tissue composition, even in the presence of temperature variations.

The concept of the model presented here is based on a previous model of fish growth [19]. Our research attempts to integrate the complete set of bodily functions (mainly growth), with inter- and intracellular regulation, to provide a multiscaled predictive tool for modeling fish in aquaculture rearing conditions. Our first-principles modeling approach includes the metabolic and regulative networks that are believed to have a large impact on growth. These are divided into functional compartments, namely, protein, lipid, and central metabolic processes, that are interconnected by mass flow. The input variables of the model are determined by the feeding process. Moreover, the output variables, i.e., the quantity of body lipids, proteins, carbohydrates, oxygen, CO_2_, and NH_3_, are all measurable (in principle), providing an experimental basis for model validation. The model setup was data-driven; the level of detail was chosen such that the values for model parameters were either known from the literature, or they could be estimated from available experimental data. In other words, almost all model parameters were identifiable. The parameters that had not been reported in the literature or that could not be estimated from experiments, particularly parameters involved in intracellular processes, were mainly estimated from data for other species of fish, or from mammal or yeast cell data. These processes (such as the tricarboxylic acid (TCA) cycle and AA catabolism) are, however, fundamentally similar in most species. As a consequence, like other models developed for similar purposes (such as the model for antibody production, see, e.g., [20,21]), the overall model includes mechanistic descriptions at the level of chemical reactions (called a 'structured model' in [20]) as well as phenomenological functions (such as Michaelis-Menten and Hill-type kinetics describing cooperative enzymes), summarizing complex regulatory pathways that are not known in detail. Ho et al. [20] refers to the latter as an 'unstructured model'.

Here, we extended the model in [19] in several respects. First, we included the experimentally reported temperature dependence of protein metabolism (protein synthesis and degradation) in the modeling framework. To the best of our knowledge, the effect of temperature was modeled relatively accurately for the first time in this type of model. We approximate the relationship between temperature and protein degradation in fish as a parabolic function. Second, we integrated several feedback control mechanisms, such as ATP-regulated fluxes in the TCA submodel, to account for the maintenance of constant energy ATP/ADP ratios. Third, we accounted for the channeling of AAs produced by protein degradation into re-synthesis in the protein metabolism cycle. Homeostasis in the previous model was achieved by two functions, lumping together the complete regulatory mechanism of the TCA cycle. This, however, was highly inaccurate, and resulted in a large discrepancy in the rapid dynamics that produced strong oscillations in the long-term dynamics (see Figure 5 in [19]). To counter the problem of strong oscillations, the model was adapted to include most of the known regulatory mechanisms in the TCA cycle [22], particularly the effect of the *α*-ketoglutarate dehydrogenase and the pyruvate carboxylase enzymes. Moreover, several regulation mechanisms involved in protein metabolism were included in the new model. They allowed for a better description of the AA pathways, improving both long-term (muscle buildup) and short-term (energy accumulation due to AA breakdown) predictions. These modifications resulted in increased accuracy of the short timescale dynamics, and, consequently, more stable long-term predictions. We provide comparisons with experimental data on the long timescale of several months for overall growth and body composition, and on a shorter time scale of hours for the regulation of energy balance and AA dynamics.

The simulation results show that our model accurately predicts growth and body composition over a long time period of several months. Furthermore, despite the difficulties involved, we demonstrate that multiscale models are able to efficiently predict many dynamic properties for a wide variety of conditions (for instance, AA retention and the effect of AA profiles, fatty acids (FAs), and glucose on growth and body composition).

In comparison to the previous model, the refined model gives qualitatively and quantitatively more accurate predictions of whole body growth, especially under widely fluctuating temperature conditions. Due to improved regulation on the short timescale (i.e., the TCA cycle rate), energy balance is much better captured, which is also reflected in better and more stable predictions on the long time scale. As a consequence, we were able to make accurate predictions over a timespan of 5-6 months, whereas the old model only gave useful results for a span of less than two months. We also observed an improvement in the prediction of AA retention. Finally, we mention here that marine fish appear to be an important future source of food. Thus, small increases in the accuracy of predictions, by only a few percent, can already assist in finding optimal feeding strategies that will have large economical and environmental consequences.

## Results

### The long timescale: Mass and body composition

**Figure 1:**
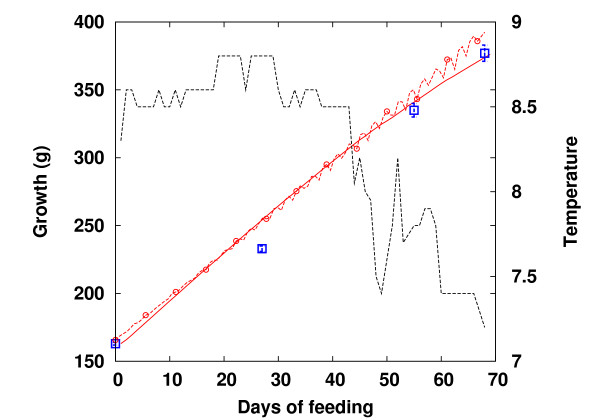
**Comparison to experiments**. Prediction of growth under conditions of varying temperature: (a) Simulation of growth in the new model (red solid), old model (red dashed circles), and experimental data (circle dashed) with the FPH15 diet given every 24 hours over the course of 68 days [23]. The black dashed curve indicates the temperature fluctuations encountered during the experiments.

**Figure 2:**
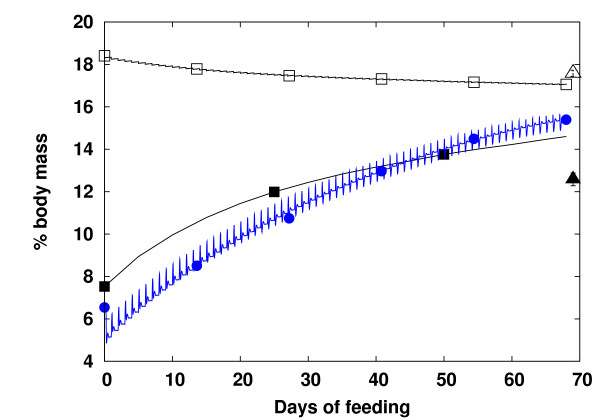
**Body composition**. Body composition (% total wet body mass) with the FPH15 diet given every 24 hours over the course of 68 days. Simulated crude protein (□) and lipid (■) in the new model compared to lipid (●) in the old model. Experimental data on protein and lipid [23] are indicated by (△) and (▲), respectively.

The model was tested against experiments with diets containing different AA profiles and fat compositions [23]. Figure 1 shows that the growth curves predicted from the previous model [19] (red dashed circles) and the revised model (red solid lines), for a timespan of 68 days, with feeding of the FPH15 diet every 24 hours, and at slightly varying temperatures, are close to the experimental data (squares). The body mass prediction was less accurate during the first 28 days of feeding, with a prediction error of about 10% for both models. This discrepancy probably arose from the lack of acclimation of the fish to the experimental conditions [23]. However, in the new model, the error was diminished to approximately 2% after 68 days, which is well within the bounds of the measurement error for all diet compositions. Growth was overestimated in the old model, leading to a bias of 35 g at the end of the simulation period. This discrepancy was due to the decreasing temperatures during the last 30 days of the experiment, and the behavior was better captured by the revised model. Moreover, due to improved regulation, the artificial strong growing daily fluctuations in the old model have disappeared in the new model. We also compared the body composition simulated in the new model with predictions from the old model for the same feeding scenario. Results are shown in Figure 2. Prediction of protein levels (square) closely followed the experimental data (triangle), whereas the prediction of lipid levels from the new model (black square) was overestimated by ~2% in comparison to the experimental data (black triangle). We note that the lipid content in the old model was calculated by estimating the protein levels as the sum of AAs, because crude protein was not available in that model. We also note that the data on fat composition from [23] was only a calculated estimation, and the real value may be higher.

**Figure 3:**
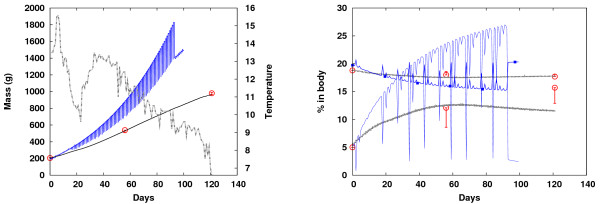
**Long-term predictions**. Long-term prediction: Body mass (left) and body fat and protein (right) over a timespan of 121 days with strong temperature variations (left, gray dotted); two different feeding strategies are shown for the new model (black curves), the model from [19] (blue curves), and experimental results (circles).

**Figure 4:**
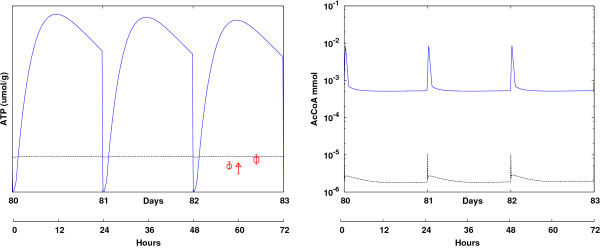
**Short timescale regulation**. Short timescale regulation: ATP levels (left) and AcCoA levels (right) simulated using the same conditions as in Figure 3, presented here for days 80-83 (given also by hours) for the new model (black dashed curve), the old model [19] (blue curve), and experimental results (circles [24], squares [25], diamonds [26]). Due to improved regulation in the TCA cycle, energy homeostasis (constant ATP levels) is predicted more accurately in the new model (black dashed curve) compared to the old one (blue curve).

The model was compared to a large scale experiment (BioMar Norway, unpublished data) in which 2500 Atlantic salmon (*Salmo Salar*) were fed standard commercial diets from July to December 2007 under strong ambient temperature fluctuations. Measurements were taken at days 0, 29, and 121, including weight and body composition. Here, the differences between the two models became even more apparent (Figure 3). High temperatures during the first 50 days resulted in a high rate of feed intake (as measured in the experiments) and excessive nutrient mass accumulation. Consequently, the old model predicted increasing oscillations, first at short timescales (ATP, TCA variables in Figure 4) and later at long timescales (growth), until an instability occurred at day 95. The new model, however, managed to handle the large nutrient intake in a stable manner. While both the final mass and the protein content were almost perfectly predicted in the new model, the results were much less accurate for the old model, particularly for fat deposition (a large discrepancy existed), probably due to incorrect temperature dependence in the model as well as the numerical instabilities. The mass was overestimated by ~50%. Underestimation of the fat content in the new model (Figure 3, right), towards the end of the simulations, may indicate faulty fat deposition dynamics at low temperatures (below 10°C).

### The short timescale-Energy balance and amino acid dynamics

Figure 4 shows the behavior of ATP and acetyl-CoA (AcCoA) for the second experiment over 120 days (as explained in Figure 3). ATP concentrations were highly unstable in the old model, oscillating between values of 0 and 40 *μ*mol/g, probably due to faulty regulation of the TCA cycle. Energy balance was much improved in the new model, with ATP values that varied slightly within tight bounds of 7-9 *μ*mol/g. This value was slightly higher than the data reported in several experiments [24-26], but the model was not parametrized under the exact conditions in which these experiments were conducted. The response of AcCoA was greatly improved between the models, with smaller oscillations after each feeding instance (note the logarithmic scale). Data on mitochondrial AcCoA from experiments with fish under similar conditions was not available.

**Figure 5:**
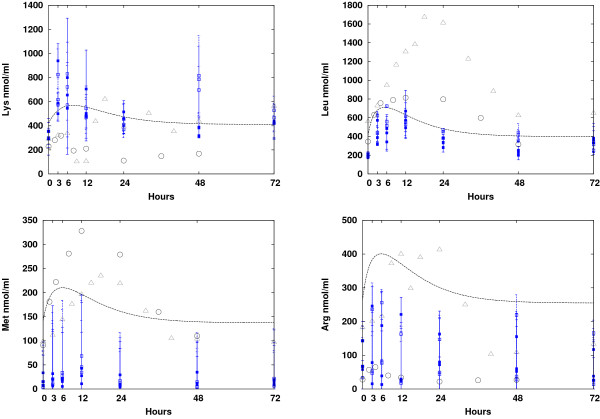
**Short timescale dynamics**. Short timescale dynamics: Comparison of simulated concentrations of several essential AAs with measurements from experiments on salmonids (rainbow trout) [27] (circles), experiments consisting of feeding with casein and AA diets [29] (full and empty squares, respectively), and casein-only diets [28] (triangles).

The prediction of essential AA dynamics was compared to experiments in which rainbow trout (family of salmonids) were fed commercial diets followed by short periods of starvation [27-29]. Results are shown in Figure 5. We observed that the predicted dynamics of some AA concentrations (only lysine, leucine, arginine, and methionine are presented here) were generally in good agreement with the experimental results. Lysine, which is considered the limiting AA in salmonids, showed levels that were well within the boundaries of the data reported by [28] and [29]. This is important because a bias in the limiting AA has a strong effect on muscle buildup and growth. However, we also note that [27] reported much higher lysine concentrations. The steady-state level of arginine was slightly overestimated. Peaks of leucine and methionine levels were smaller than the experimentally measured levels, but the steady-state AA levels were better captured for these two than for arginine.

These discrepancies could have several sources. For example, the AA requirement profiles of the two species (salmon and trout) that were compared for protein building were different. These profiles are inserted into the model in the profile filter of the protein metabolism model (see the Protein Metabolism section). Unfortunately, we do not have the time-series data available for salmon. Another source of the discrepancy could be the composition and consistency of the diets used in the experiments. These diets can vary with respect to protein type (for instance the protein casein versus free AA diets, as in [28], or the commercial diet versus crystalline AA diets [27]), fat, and carbohydrates, as well as with respect to the processing to which the ingredients were subjected [12]. These differences (diet ingredients, formulation, and processing) have a large effect on the digestion and absorption of the nutrients in the gut, particularly on the AAs. It is likely that the concerted effect of these unmodeled factors resulted in the observed experiment-to-experiment variation. Further investigation is necessary.

We also mention that the predicted peak values occurred earlier (5-10 hours) than those reported in the experiments (12 h) for almost all AAs. Two factors could explain this deviation: Nearly all fish in the experiments were force-fed and, therefore, stressed, which has a strong impact on the absorption and digestion rates of nutrients [30]. Additionally, although the metabolic model presented here may have been accurate, the digestive model may have been too simple. The simplified digestive model used here did not account for many of the interactions between nutrients, e.g. competition of the AAs for the carriers during absorption, rate of digestion of the diet, nutrient breakdown in the gut due to enzyme interactions, density of the commercial diets as a result of processing, the quantity and type of lipids in the diet, and the general effects of lipids on absorption. All of these unmodeled factors (and other potential factors, listed in [12,30]) may have resulted in earlier AA absorption, and, consequently, earlier simulated peaks compared to the experiments. A more comprehensive model of the digestive system is under preparation in order to increase the accuracy of the AA metabolism model.

Although not originally in the scope of this model, the short-term predictions (AA, ATP, ADP, and TCA components) were reasonably captured, in spite of the unmodeled dynamics. Because short-term and long-term predictions are interconnected (e.g. ATP regulates growth, and protein buildup is linked to the AA metabolism), a serious deviation in either of the timescales would be a matter of concern, as it would lead to faulty predictions on both timescales. By capturing the short timescale correctly, the model assures the credibility of the longer timescale.

### Effect of temperature

**Figure 6:**
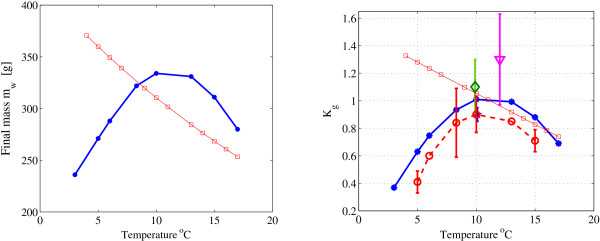
**Effect of temperature**. The effect of temperature after 68 days using the FM diet [23]: (a) Prediction of body mass as a function of temperature for the new model (blue dots) and the old model [19] (red squares). (b) Protein turnover rates *K*_*g *_taken from: New model simulations (blue solid dots), previous model (red squares), [15] (dashed), [59] (triangle at 12°C), and [31] (diamond at 10°C), as a function of temperature.

Figure 6 shows the simulation results of both models over a period of 68 days, with a diet of FPH15 and temperatures between 3 and 18°C. In each simulation, the temperature was held constant. Here, the new model clearly outperformed the old model, which demonstrated an almost linear decrease of final mass and protein turnover rate with increasing temperature. In comparison, the new model response to variations in temperature not only qualitatively captured the experimental trends, but even lay within the reported experimental values. The protein turnover rate (% protein day^-1^) was calculated using the expression *K*_*g *_= 100·[ln *p*(*t*) - ln *p*(*t*_0_)]/*d*, where *t *and *t*_0 _are the final and initial time, *d *is the total time in days, and *p *is the protein mass [16]. The effect of the temperature on *K*_*g *_in the simulations was slightly higher than the temperature effect reported for Atlantic wolfish, *Anarhichas lupus *[15], but this difference was consistent with reports on Atlantic salmon [14,31]. Note, however, that the trend (shape) of the effect was preserved. Small temperature variations, particularly far from the optimal value *T*_*opt*_, considerably influenced the protein and water deposits in the body, and, consequently, the total body mass, consistent with experimental data [12].

### Amino acid retention

**Figure 7:**
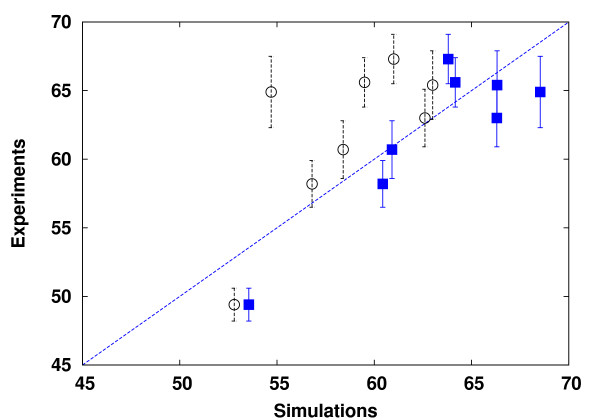
**Amino acid retention**. Deviation of the AA retention (%) in the presented model (squares), and earlier model (circles) [23]. The dashed line indicates perfect prediction.

The new model for protein metabolism allows for better prediction of AA retention, as can be seen in Figure 7. AA retention is directly affected by both protein degradation (as in [19]) and the recycling of AAs into protein synthesis. The largest improvements in AA retention modeling were reported for tyrosine (2.3% compared to 15.7%), threonine (5.3% compared to 9.3%), and phenylalanine (0.6% compared to 2.4%). The mean squares errors were 23.3 for the earlier model [19] versus 17.3 for the model presented here.

## Discussion and Conclusion

The multiscale model presented here allowed prediction of growth, body composition, and AA retention under varying temperatures for a period of several months, with relatively low deviation from the experimentally measured quantities. First-principles (mechanistic) modeling, combined with empirical observations, appears to be crucial to achieving successful predictions over long periods. Earlier models could indeed predict growth over some timescales [5,32,33], but the predictions were almost always limited to only a few properties for each model, as is common in static models, such as the regression and bioenergetic models [9]. Even dynamical models that predicted several properties simultaneously [3,4,19] could not guarantee accurate predictions for more than several weeks. Our model was able to predict over a long timespan and under variable temperatures. In this model, energy regulated most molecular processes and the flux balance between metabolic compartments (protein, lipid, and central metabolism). Maintaining homeostasis (constant steady-state ATP levels) in the model, via the regulation of metabolite fluxes from one compartment to another, assisted in the accurate prediction of growth and yielded better prediction of TAG and protein levels in tissue.

Temperature has a large impact on animal growth [12], but, to the best of our knowledge, most existing models (particularly bioenergetic models) that consider temperature only do so for a constant temperature (see, for instance, earlier dynamic models [5,19]). The complex effect of temperature on the various processes in the body, and, in particular, on the protein degradation process, is mostly obscured and cannot always be measured [15], rendering the effect of temperature on growth difficult to model. Due to its dynamical characteristics, the model proposed in this study can account for instantaneous changes in the temperature, down to a resolution of seconds. Therefore, it can be applied to systems with considerable temperature fluctuations, such as those which naturally occur when the seasons change. In the model presented here, we used the measured effect of water temperature on protein synthesis and empirical data on temperature's concerted effect on growth to estimate the effect of temperature on protein degradation and lipid metabolism. The simulation results were reassuring: the simulated total growth correlated well with the growth data from experiments, and the effect of protein turnover was accurately captured (see Figure 6). This indicated that the concerted effect of temperature, its effect on the individual processes (e.g. the turnover process), and particularly on the protein degradation were all correctly modeled. It was suggested here that temperature influences protein degradation in a parabolic manner, as described by equation (7). This hypothesis was reasonable because the consensus of experimental data (reported in the literature) suggests that protein synthesis is linearly affected by temperature [13,15,34], and growth has a bell-shaped dependency [12,15]. However, we assumed that temperature linearly affected other processes (lipid and central metabolism), which is not necessarily the case. Nevertheless, given that more than 80% of fish mass consists of protein and water [35], the remaining processes are not expected to exert a large perturbation on the overall growth model. For comparison, a linear protein degradation-temperature dependence (as modeled in [19]) appeared to yield a faulty response (Figure 6a). We emphasize that the parabolic dependence of degradation on temperature is an interesting result in its own right, which might motivate further experimental investigations.

The model described here is composed of processes that occur on very different timescales. Concentrations of TCA intermediates change within seconds and minutes (for instance Acetyl-CoA in Figure 4). AA concentrations (Figure 5), which rise rapidly within minutes of nutrient uptake, exhibit fast dynamics in the model. A large decrease in ATP concentration, within hours of feeding, occurs due to the large consumption of ATP during protein synthesis. ATP levels relax back nearly to steady-state conditions, by way of several regulatory mechanisms, after several hours (Figure 4). The consumption of AAs in response to feeding occurs on a timescale of hours. In contrast, the buildup of protein tissue and TAG reserves occurs over days and weeks, and considerable changes to the progress of growth occur over a timescale of many days or even months (Figure 1). Thus, all timescales within this wide range are relevant to model development, implying a variety of challenges for model assessment. First, errors in the short timescales of seconds and minutes can rapidly accumulate and render the long-term growth prediction unreliable, as we have seen in the previous simulations (Figures 3 and 4). Errors in the short timescales should be limited to ensure valid long timescale predictions, but the relevant intracellular variables are usually difficult to measure. Second, dimension reduction methods via separation of timescales, such as quasi-steady-state or rapid equilibrium approximations, probably fail here, because the relevant timescales vary over a wide range (Figures 4 and 5) and cannot be separated. The time needed for the ATP/ADP ratio to return to its steady-state may be longer than the time between two feeding scenarios (Figure 4), which can cause growing oscillations on the long timescale (Figure 1). Hence, we cannot apply a quasi-steady-state approximation for ATP. Third, interrelated processes with large differences in timescales usually translate into stiff differential equations, which require advanced numerical integration methods. This is also currently a limitation of the model. Because we found that implicit methods implemented in MATLAB failed here, we used a fixed step size that was manually adapted to the fast timescale, making the simulation calculations rather slow. Moreover, stiffness can lead to a variety of problems for methods such as bifurcation or sensitivity analysis. These issues constitute only some of the difficulties inherent in solving multiscale problems. Despite these challenges, omitting any of the regulatory mechanisms incorporated at different timescales (at the cellular and tissue levels) indeed degraded the prediction accuracy. We concluded, therefore, that multiscale modeling is often important for accurate predictions of complex systems.

We did not make use of any potentially available real-time measurements during long period experiments. Because fish in rearing conditions grow over a period of four years, we expect a significant discrepancy in the results, particularly because the feeding regime and composition changes during this time. Unmodeled dynamics, such as the effect of aging, are also a major concern for discrepancies when simulating long timespans (years). However, it is possible to increase the accuracy of the prediction by acquiring measurements of several variables (NH_3_, CO_2 _excretion, O_2 _consumption) and use this data to correct for discrepancies along the way. This procedure is equivalent to applying feedback from measurements in engineering control theory. It will also allow re-optimization of the feed to adjust for unmodeled variations due to, for instance, water salinity, stress in the rearing facility, and change in water pH values.

To conclude, the multiscale model presented here is able to predict growth, body composition, and AA retention relatively accurately over several months. Predictions hold even when strong temperature fluctuations occur, a key property for any model that is applied to the design of feeding experiments. We suggest that the protein degradation process in fish has a parabolic shaped dependency on temperature, but this hypothesis requires further investigation.

## Methods

Our model is founded on a previous work described in [19]. The main components of the model are the nutrients that enter the metabolic processes as AAs, glucose and fatty acids, the proteins composed of twenty AA, lipids, several metabolites of the central metabolism, and energy. In the following, we give an overview of the model, and the new components are described in detail.

Feed is transformed into a model input using the digestive model described in [36]. According to this model, digestion has a smoothing effect by translating the discontinuous feeding process into an input function that is continuous over time. The nutrients absorbed in the digestive system are transformed in the various organism compartments to other metabolites or to energy. It is assumed that body mass is mainly composed of proteins, TAG in the adipose cells, water, and minerals, and growth is modeled in the same manner as described in [19].

**Figure 8:**
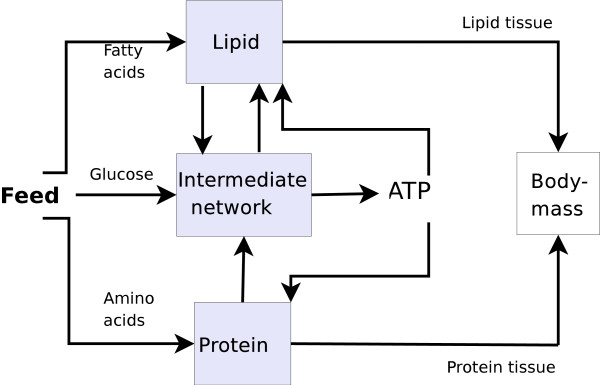
**The model**. A schematic representation of the model. The main processes in the model are lipid, protein, and TCA metabolism, which are interconnected by mass flow.

The model, presented as a set of ordinary differential equations (ODEs), consists of functional compartments interconnected by mass flow. The overall structure is shown in Figure 8. These compartments represent simplified inter- and intracellular processes that are significant for growth. The intracellular structure is composed of the cytosol and the mitochondria matrix, and transport between these is modeled as a set of first order kinetic reactions. The transformations of the nutrients in the metabolic pathways are described by stoichiometric equations. For instance, the breakdown of AAs is modeled by the chemical reaction(1)

where the stoichiometric coefficients *a*, *b*, *c*, and *d *depend on the specific AA [37]. All model states, input and output variables, and corresponding timescales are given in Table 1.

**Table 1:** Model overview.

Submodel	Variables	Interpetation	Dim.	Eqn.	Param.	timescale
Protein	*a*_*a*_	Free amino acids (AA) concentration	ℙ^20^	Table 2	Table 3	medium
	*a*_*p*_	Amino acids channeled to protein synthesis	ℙ^20^			medium
	*a*_*b*_	Amino acids channeled to catabolism (break-down)	ℙ^20^			medium
	*p*_*a*_	Tissue protein, given by its amino acids composition	ℙ^20^			slow

Lipid	AcCoA_*cyt*_	Acetyl-CoA in the cytosol	ℙ^1^	Table 4	Table 5	fast
	malCoA	Malonyl-CoA	ℙ^1^			fast
	FA	Fatty acids (palmitate)	ℙ^1^			medium
	TAG	Tri-acylglycerol	ℙ^1^			slow

Intermediate metabolism	pyr	Pyruvate	ℙ^1^	Table	Table 8	fast
				6, 7		
	AcCoA	Acetyl-Coa (matrix)	ℙ^1^			fast
	cit	Citrate concentrations in the mitochondria	ℙ^1^			fast
	iso	Isocitrate concentrations in the mitochondria	ℙ^1^			fast
	keto	*α*-ketoglutrate concentrations in the mitochondria	ℙ^1^			fast
	succ	Succinate concentrations in the mitochondria	ℙ^1^			fast
	mal	Malate concentrations in the mitochondria	R^1^			fast
	oxal	Oxaloacetate concentrations in the mitochondria	ℙ^1^			fast
	mal_*cyt*_	Malate concentrations in the cytosol	ℙ^1^			fast
	oxal_*cyt*_	Oxaloacetate concentrations in the cytosol	ℙ^1^			fast
	Glu	Glucose concentrations (glycolysis process)	ℙ^1^	Table 9	Table 9	fast
	g6p	glucose-6-phosphate concentrations (glycolysis)	ℙ^1^			fast
	pGt	6-phosphogluconate concentrations (glycolysis)	ℙ^1^			fast
	G3p	glyceraldehyde 3-phospate concentrations (glycolysis)	ℙ^1^			fast

(feed)	*u*_feed_	The nutrients FA, glucose and 20 amino acids consumed	ℙ^23^	N/A	N/A	medium
Output	*y*(*t*)	The output of the model, consists of protein (sum of its amino acids), storage lipids (TAG), NH_3_, O_2 _and CO_2_, all in units of gram	ℙ^5^	N/A	N/A	f/s/m

The model states, input and output variables and its respective timescales. The underscore *cyt *denotes variables in the cytosol. Amino acids (AA) vectors consist of the 20 amino acids. All units are given in [*μmol*] unless stated otherwise.

In the following, we describe the simplified processes of the functional compartments and the regulation of the mass flux between these compartments in more detail.

### Mass flux regulation

The velocity of reactions driven by important regulatory enzymes is described by a Hill-type kinetic model [38,39],(2)

for activation and inhibition, respectively. *V *denotes the maximal reaction velocity, the exponent *β *is the Hill coefficient, and  is the half-saturation value. We also introduce a second phenomenological regulatory function into the model as the empirically observed upper buildup capacity for protein deposition in fish [35]. This is described by a simple piecewise linear function(3)

with threshold value *x*_*c*_.

### Protein metabolism

**Figure 9:**
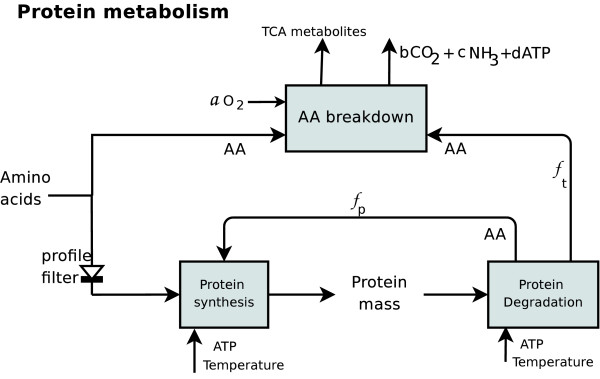
**Protein metabolism sub-model**. Overview of the protein metabolism model with arrows describing material flow. AAs are used for protein synthesis in a specific composition satisfying the profile of the tissue protein. Remaining AAs are broken down into TCA metabolites and enter the intermediate metabolism. Protein synthesis and degradation are energy and temperature dependent. The fraction *f*_*p *_of AAs emerging from protein degradation is reused for protein synthesis, and the fraction *f*_*t *_= 1 - *f*_*p *_is broken down.

For simplicity, proteins are described by a single representative tissue protein, which is composed of a specific AA profile. The model for protein metabolism is shown in Figure 9 and consists of a protein synthesis model, protein degradation model, and AA regulation. The protein synthesis submodel utilizes as many free AAs (essential and non-essential) as possible that enter the protein metabolism compartment. We denote this fraction of AAs by *a*_*p *_(Table 2). Because the composition of these AAs must meet the tissue protein profile (indicated by the profile filter in Figure 9), all remaining AAs are directly channeled from the AA pool to catabolism, are deaminated, and are broken down into ketone groups. The protein synthesis and degradation submodels require ATP and depend on temperature. AAs emerging from protein degradation are reused for protein buildup or channeled through catabolism. Both pathways are assumed to be entered with constant fractions *f*_*p *_and *f*_*t *_= 1 - *f*_*p*_, respectively. Whereas the previous model contained a complex synthesis model and a simple linear relationship between degradation and synthesis submodels, here we simplified the synthesis model significantly, revised regulation, and revised the interconnection between submodels. The equations and parameter values are provided in Tables 2, 3.

**Table 2:** Protein metabolism - equations.

State variables	
*a*(*t*) = (*a*_*a*_, *a*_*p*_, *a*_*b*_, *p*_*a*_)^*T*^	
*a*_*a *_= amino acids in the free AA pool	
*a*_*p *_= amino acids channeled to protein synthesis	
*a*_*b *_= amino acids channeled to catabolism	
*p*_*a *_= tissue protein given by its amino acid composition	
*u*_*aa *_= 20 amino acids that are recieved from the feed (input variable)	

**State equations**	

= -*k*_*aa*_*a*_*a *_- *U*_*ps*_*k*_*p*_*a*_*a *_+ *u*_*aa*_	*k*_*aa *_= 1/s
= *U*_*ps*_*k*_*p*_*a*_*a *_- *v*_*s*_(*t*)*a*_*p *_+ *f*_*p*_*v*_*d*_(*t*)*p*_*a*_	*k*_*p *_= 1/s
= *k*_*aa*_*a*_*a *_+ *f*_*t*_*v*_*d*_(*t*)*p*_*a *_- *a*_*b*, *cat*_	
= *v*_*s*_(*t*)*a*_*p *_- *v*_*d*_(*t*)*p*_*a*_	

State vector *a*(*t*) and equations for the protein metabolism submodel illustrated in Figure 9.

**Table 3:** Protein metabolism - parameters.

Parameter	Description	Value	Source
*f*_*p*_, *f*_*t*_	Flux of amino acids post degradation process	0.75, 0.25	[23,60]
*T*_*opt*_	Optimal operation temperature	12°C	[12]
*ϑ*_*s*_	Effect of temperature on protein synthesis rate	1/16	[13]
*ϑ*_*d*_	Parabolic temperature-degradation constant	0.013/°C^2^	[15]
*b*_*d*_	Minimum degradation rate factor in *f*_*d*_	0.052	[13]
*ψ*	Genetically determined efficiency of rate of protein degradation	0.2 ~0.85	assumed
	Concentrations of EAA in the pool	*μ*mol/g	[61]
*p*_*c*_	Protein buildup capacity constant	0.185	[35]
*c*_*d*_	Effect of EAA on protein degradation	2	assumed
*V*_*ps*_	Gain on the protein buildup capacity. Estimated from large scale experiments and simulations, not a sensitive parameter [62]	10	estimated
*V*_*pd*_	Gain on the protein degradation. Estimated from simulations of nitrogen excretion and Comparison with data [34]	10	Estimated

Parameters associated with the protein metabolism process.

#### Synthesis model

**Figure 10:**
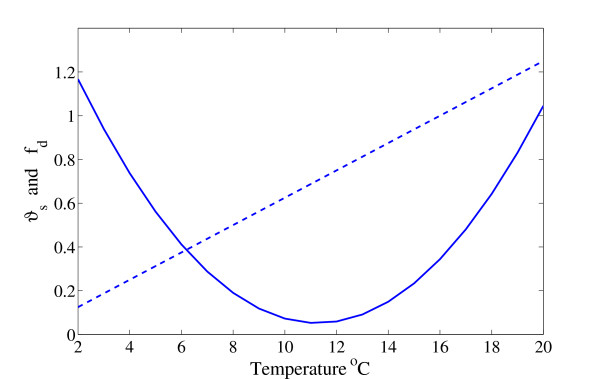
**Modeled temperature**. Modeled temperature-dependent prefactors of protein synthesis (dashed line, equation 4) and degradation (solid line, equation 7).

The rate of protein synthesis (denoted *v*_*s*_(*t*)) is modeled using simplified Michaelis-Menten kinetics, affected by temperature and availability of energy. The effect of temperature on growth, metabolic rates, and protein synthesis has been studied in fish in general [40], and in Atlantic salmon [13] and Atlantic wolfish [15] in particular. The rates of white muscle and whole body protein synthesis were observed to increase linearly between 5 and 14°C. Based on these studies, the effect of temperature *T *on the translation rate is modeled by a simple linear equation, as shown in Figure 10, with a slope parameter *ϑ*_*s*_(*T*) that increases linearly with temperature,(4)

Here, *π*(*t*) is the peptide chain synthesized at time *t*. Assuming no cellular stress is introduced (for instance, by a virus attack), the calculation of the synthesis rate  can be reduced to include only a simplified eukaryotic initiation factor (eIF)-2 regulation model. Here, we refer to [41] for further details. This reduction makes the calculations more efficient and eliminates uncertainties associated with unknown parameters in the full-scale protein synthesis initiation submodel [42]. In contrast to the previous model [19], here, the full-scale submodel was reduced to a simple set of reactions transforming the sequence of AAs into proteins using dynamics similar to Michaelis-Menten kinetics. The rate of protein synthesis is given by(5)

where *U*_*ps *_denotes the regulation of the buildup protein capacity described below, and  is the flux of AAs available from protein degradation.

#### Degradation model

The protein degradation process is described as (*t*, *T*) = *v*_*d*_(*T*)*p*_*a*_(*t*). Here *v*_*d*_(*T*) is a variable degradation factor that depends on temperature and is regulated by free essential AAs (EAAs). Temperature affects protein turnover (defined as protein synthesis-degradation) and degradation of the whole body and the whole muscle in Atlantic salmon and Atlantic wolfish in a nonlinear manner [12,13,15]. Hence, we improved the model accordingly by taking this nonlinearity into account. Using data from [12,13,15], we re-modeled the temperature effect on degradation rate, empirically, through the function *v*_*d*_(*T*),(6)

where *ψ *is the rate of peptide targeting and breakdown, *c*_*d *_is a constant (described below), and *U*_*pd *_describes the effect of EAA on degradation rate (described below). The parameter *ψ *might be genetically determined [16] with variations of up to 20%. Thus, this parameter must be estimated for a specific fish species. Temperature is known to have an optimal value *T*_*opt *_for growth; hence, we assumed that the function has a parabolic shape, at least locally about *T*_*opt*_. The simplest way to describe this assumption is to use a second order equation,(7)

with *T*_*opt *_= 11.8°C [12]. The parameters *ϑ*_*d *_= 0.013 and *b*_*d *_= 0.052 were empirically determined according to [15].

#### Regulation of protein metabolism

Regulation in the protein metabolism model is performed by modulating both the flow of AAs *U*_*ps *_and protein degradation *U*_*pd*_. Protein deposition in fish has an upper buildup capacity, which was found empirically to be approximately 18-19 g protein per 100 g body weight, depending on size and age [35]. As protein deposition approaches its capacity, further synthesis is inhibited. This empirical evidence for upper protein capacity has not yet been fully explained and might incorporate several regulatory steps in cell growth. The upper capacity is modeled here by the piecewise linear regulation function, *U*_*ps *_= *U*^*pl*^(*p*(*t*); *p*_*c*_*m*_*w*_(*t*)).

Deficiency in the EAAs promotes protein degradation that supplies the missing EAAs from the body tissue.

This suggests a Hill-type model, *U*_*pd *_= *U*^*i*^(EAA_*j*_; *V*_*pd*_, *β*_*a*_, ), with  being the minimum required concentrations of the *j *∈ [1, 2, ..., 20] intracellular EAAs (given, for instance, by [35,43]). Because we assume the protein degradation process to be irreversible, the rate *v*_*d*_(*T*) in (6) must always be positive, and the value of *c*_*d *_should satisfy *c*_*d *_> *V*_*pd *_≥1. Furthermore, the values for the parameters *c*_*d *_and *V*_*pd *_must be assumed because the regulation *U*_*pd *_is not associated with any specific enzyme but rather represents the cumulative effect of degradation regulation. However, we tested several values and observed no significant differences in our results. The model appears to be robust with respect to changes in these values.

### Lipid metabolism

**Figure 11:**
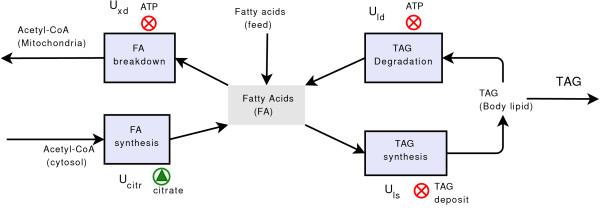
**Lipid metabolism sub-model**. Schematic drawing of lipid metabolism. Cytosolic AcCoA entering the lipid metabolism compartment from the intermediate metabolism is used for FA synthesis. FAs are either broken down, leading to AcCoA in the mitochondria, which fuels the TCA cycle, or are used for TAG synthesis, thereby increasing the body weight. ATP inhibits TAG degradation and FA breakdown. TAG deposit levels inhibit (⊗) TAG synthesis, and citrate levels in the mitochondria promote () FA synthesis.

The lipid metabolism model, shown in Figure 11 and described in Table 4, incorporates FA synthesis and oxidation, and TAG synthesis and breakdown. The principal structure of this submodel remains similar to the previous model. However, both the metabolite source of the submodel (namely AcCoA) and the regulations were revised. To simplify the model, palmitate is incorporated as the sole FA involved in lipogenesis. We further assume that glycerol is not rate-limiting in TAG synthesis [44], and that CO_2 _levels are abundant. AcCoA is transported out of the mitochondria using the tricarboxylate carrier. This carrier system is modeled here using two separate variables for the metabolite, AcCoA in the mitochondria and cytosolic AcCoA (denoted AcCoA_*cys*_). The latter is transformed into malonyl-CoA by the cooperative enzyme acetyl-CoA carboxylase, then to palmitate by the multi-enzyme complex fatty acid synthase.

**Table 4:** Lipid network states and parameters.

State space equations
*d*(AcCoA_*cyt*_)/*dt *= -7k_*q*1_AcCoA_*cyt*_·ATP-*k*_*q*2_AcCoA_*cyt*_·malCoA·NADPH
*d*(malCoA)/*dt = 7k*_*q*1_AcCoA_*cyt*_·ATP - 7*k*_*q*2_AcCoA_*cyt*_·malCoA·NADPH
*d*(FA)/*dt = k*_*q*2_AcCoA_*cyt*_·malCoA·NADPH - 3*U*_*ls*_*k*_*ls *_FA + 3*U*_*ld*_*k*_*ld*_TAG - *U*_*xd*_*k*_*xd*_FA·NAD^+ ^+
*d*(TAG)/*dt = U*_*ls*_*k*_*ls*_FA - *U*_*ld*_*k*_*ld*_TAG

The state space equations and the parameters of lipid metabolic network. AcCoA_*cyt *_is the concentration of acetyl-CoA in the cytosol. malCoA is the concentration of malonyl-CoA.

#### Regulation of lipid metabolism

Cytosolic AcCoA is carboxylated to malonyl-CoA, for which citrate is an allosteric activator of the cooperative enzyme AcCoA carboxylase. AcCoA carboxylase affects FA synthesis in a manner that can be described by the Hill equation [45-47], here described by *U*_*fs *_= *U*^*a*^([citr]; *V*_*fs*_, *β*_*fs*_, ·*m*_*w*_) with empirically determined constants (Table 5). Experiments on pigs and rats, and reports from humans, showed that utilization of AcCoA for FA synthesis is low [48,49]. We assume that fish synthesize FA only in cases of exceedingly high citrate levels.

**Table 5:** Lipid metabolism - parameters.

Parameter	Description	Value	Source
k_*q*1_, k_*ls*_, k_*ld*_	rate constants for the lipid metabolism reactions	1, 1, 0.1/*μ*mol/s	assumed
k_*q*2_, k_*xd*_	rate constants for the lipid metabolism reactions	100, 0.3/*μ*mol^2^/s	assumed
V_*fs*_	maximal velocity of FA synthesis, estimated to produce rapid Hill reaction, according to [45]	60	estimated
*β*_*fs*_	Hill coefficient FA synthesis	3	[45]
	citrate concentration at half FA synthesis reaction	0.2 *μ*mol/g	[45]
	capacity of TAG deposition in the body	30%	[63]
*β*_*ls*_	exponential coefficient for TAG deposition, estimated from simulations producing slow Hill dynamics	2	estimated
*V*_*ls*_	gain of the lipid synthesis function, estimated from simulation	1*μ*mol	estimated
*β*_*xd*_	Hill coefficient of the *β*-oxidation process	4	assumed
	concentration of ATP at half saturation of *β*-oxidation process	7 *μ*mol/g	[25,64,65]
V_*xd*_	maximal velocity of *β*-oxidation	0.3 *μ*mol	[53]
*β*_*ld*_	Hill coefficient of TAG degradation. Estimated from [25,66] and simulations to maintain reasonable Hill dynamics	4	estimated
	concentrations of ATP at half saturation in the TAG degradation process	7.8*μ*mol/g	[25,66]
V_*ld*_	maximal velocity TAG degradation. Estimated from [25,66] and simulations to maintain reasonable Hill dynamics	10*μ*mol	estimated

Parameters in the lipid metabolism model.

There is strong evidence for a mechanism known as *lipostat *in fish, in which TAG deposition does not exceed an upper limit defined relative to body mass [50,51]. However, the regulatory mechanism responsible for this phenomenon is currently unknown. Thus, regulation is modeled here using the following saturation function [51]:(8)

where the constant  is the capacity of a TAG deposit (percent of the whole body), ℳ_*TAG *_is the molecular mass of TAG, and *V*_*ls *_≥1 is the maximal reaction velocity.

The rate of the *β*-oxidation process is regulated at several levels. Although a carnitine shuttle appears to be an important regulatory step for *β*-oxidation in the liver [52], it is not as important in skeletal muscles and is not modeled here. During muscle contraction, for which the primary function of *β*-oxidation is the provision of energy, the *β*-oxidation flux is regulated through the ATP levels and the ratio of NAD^+ ^to NADH [52,53]. ATP regulation of the process is modeled via *U*_*xd *_= *U*^*i*^([ATP]*m*_*w*_; *V*_*xd*_, *β*_*xd*_, *m*_*w*_), where  is the ATP level at half saturation, which varies between 6 and 10 *μ*mol·g^-1^, depending on the cell type (Table 5). The rate of TAG hydrolysis is regulated similarly to the regulation mechanism described in equation (8). The function *U*_*ld *_is assigned a slightly lower *K*_*A *_value (i.e.,  in Table 5).

### Intermediate metabolism

#### TCA cycle

**Figure 12:**
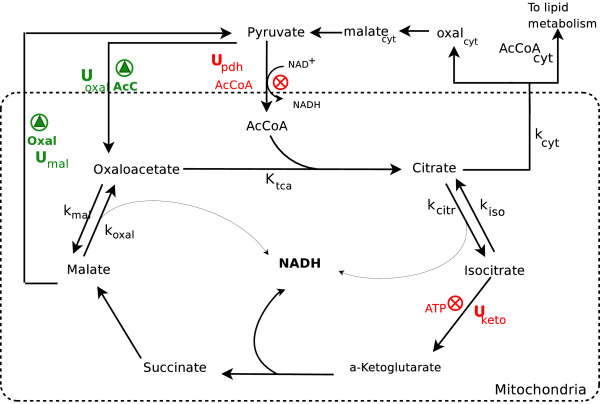
**Central metabolism sub-model**. Regulation in the tricarboxylic acid (TCA) cycle model. Intermediates from AA catabolism enter the cycle, leading to NADH production. *U*_*i *_represent regulatory functions. *U*_pdh _and *U*_keto _are inhibited (⊗) by ATP, whereas *U*_oxal _and *U*_mal _are promoted () by AcCoA and oxaloacetate, respectively.

The TCA cycle submodel in Figure 12 constitutes the core of the model. Mass flow into this submodel includes intermediates from AA catabolism, AcCoA from oxidation, and pyruvate from glycolysis. The TCA cycle is composed of a set of reactions (Table 6) with the purpose of creating ATP. The most important improvement, relative to the old model, was the regulation of the TCA rate. Here, this was performed by a complex set of enzymatic regulatory mechanisms.

**Table 6:** Reactions of the central metabolism submodel.

pyruvate + NAD^+^		Acetyl-CoA+ NADH
Acetyl-CoA + Oxaloacetate		citrate
citrate + NAD^+^		*α*-ketoglutarate +NADH + CO_2_
*α*-ketoglutarate + NAD^+^		succinate + NADH + CO_2_
succinate + ADP^+^		fumarate+ATP
Fumarate		malate
malate + NAD^+^		oxaloacetate+NADH
malate		pyruvate

In this submodel, we assume the following: (1) Water, as precursor, is not considered, and (2) Guanosine di- and triphosphate (GDP and GTP) are interchangeable with ADP and ATP, respectively, because they are nearly equal in terms of energy cost and gain. The equations and parameters for the cycle are given in Tables 7 and 8, respectively. The first regulatory step is the activation of pyruvate dehydrogenase (PDH), an enzyme complex that allosterically regulates pyruvate transformation to AcCoA. Levels of the latter inhibit regulation in a complex manner [54,55]. Regulation is modeled here by *U*_pdh _= *U*^*i*^([acc]; *V*_pdh_, *β*_pdh_, *m*_*w*_), where  is the level of AcCoA at half saturation.

**Table 7:** Central metabolism equations.

State space equations	
= *h*_2 _+ *h*_3 _- *h*_1_- *h*_4_	= *h*_10 _- *h*_11_
= *h*_1 _- *h*_5_	= *h*_11 _+ *h*_12 _- *h*_13 _- *h*_3_
= *h*_5 _+ *h*_7 _- *h*_6_- *h*_8_	= *h*_13 _+ *h*_4 _- *h*_12 _- *h*_5_
= *h*_6 _- *h*_7 _- *h*_9_	= *h*_15 _- *h*_2_
= *h*_9 _- *h*_10_	= *h*_8 _- *h*_15_

**Network functions**	

*h*_1 _= *U*_pdh_*k*_*r*1 _[pyr]·[NAD^+^]	*h*_9 _= *U*_keto_*k*_*r*9 _[iso]·[ATP]
*h*_2 _= *k*_*r*2 _[mal_*cyt*_]·[NADP^+^]	*h*_10 _= *k*_*tca *_[keto]·[NAD^+^]
*h*_3 _= *U*_mal_*k*_*r*3 _[mal]·[NAD^+^]	*h*_11 _= *k*_*citr *_[suc]
*h*_4 _= *U*_oxal_*k*_*r*4 _[pyr]·[ATP]	*h*_12 _= *U*_mal_*k*_mal _[oxal]·[NADH]
*h*_5 _= *k*_*tca *_[AcCoA]·[oxal]	*h*_13 _= *k*_oxal _[mal]·[NAD^+^]
*h*_6 _= *k*_*citr *_[cit]	*h*_14 _= *U*_oxal_*k*_*r*1 _[pyr]·[ATP]
*h*_7 _= *k*_*iso *_[iso]	*h*_15 _= *k*_*r*2 _[oxal_*cyt*_]·[NADH]
*h*_8 _= *k*_*cyt *_[cit]·[ATP]	

State variables *r*(*t*), state space equations of intermediate metabolism (TCA cycle) and the reactions *h*_*i*_

**Table 8:** Central metabolism - parameters.

Parameter	Description	Value	Source
*k*_*r*1,3,4,9_	rate constants for the network functions *h*_1_, *h*_3_, *h*_4 _and *h*_9_	1/*μ*mol^2^/s	assumed
*k*_*r*2_	rate constant for the network function *h*_2_	1/*μ*mol/s	assumed
V_pdh_	maximal velocity of the reaction involving pyruvate dehydro-genase (PDH)	25*μ*mol	[67]
	concentrations of AcCoA at half saturation of the reaction involving PDH	0.2*μ*mol	[67]
V_keto_	maximal velocity of isocitrate dehydrogenase reaction	100*μ*mol	[68]
	ATP levels at half saturation of the isocitrate dehydrogenase reaction	7*μ*mol/g	[25,66,69]
V_mal_	maximal velocity of the malate-pyruvate pathway, must be smaller than k_oxal _to maintain regulation. Estimated from [70]	0.1 *μ*mol	estimated
	oxaloacetate levels at half saturation of the malate-pyruvate pathway	0.04 nmol/g	[71]
V_oxal_	maximal velocity of the pyruvate-oxaloacetate pathway	5 *μ*mol	[58]
	AcCoA levels at half saturation in the pyruvate-oxaloacetate pathway	3*μ*mol/g	[58]
*k*_*tca*_	rate constant in the TCA cycle	10/s/*μ*mol	[69]
*k*_*citr*_	Half saturation constant in citrate aconitase reaction	1.4/s	[72]
*k*_*iso*_	rate constant of citrate aconitase reversible reaction	10/s	[73]
*k*_oxal_	rate constant of malate to oxaloacetate reaction	1.4/s/*μ*mol	[70]
*k*_mal_	rate constant of oxaloacetate to malate reaction	2.8/s/*μ*mol^2^	[70]
*k*_*cyt*_	rate constant of citrate transport from the matrix to the cytosol (by tricarboxylate carriers), estimated from [74], must be slower than isocitrate dehydrogenase reaction	1/s/*μ*mol	estimated

Constants associated with the intermediate metabolism submodel

The second regulatory step in the TCA submodel regulates the rate of oxidation of isocitrate to *α*-ketoglutarate via the allosteric enzyme isocitrate dehydrogenase. This enzyme is composed of three distinguishable subunits [56,57], suggesting the description, *U*_keto _= *U*^*i*^([ATP]; *V*_keto_, *β*_keto_, *m*_*w*_). This suppresses the flux of isocitrate to *α*-ketoglutarate when energy is abundant, i.e., ATP ≫ *m*_*w*_. The reversible reaction to citrate enables citrate accumulation and transport from the matrix to the cytosol to participate in the FA synthesis process. *V*_keto _is a sensitive parameter, because the equilibrium may not tend towards *α*-ketoglutarate if ATP is deficient.

Additionally, two regulatory functions preserve homeostasis in the TCA cycle. These regulate the concentrations of malate and oxaloacetate to prevent excessive accumulation of intermediates. In the malate-pyruvate pathway (Figure 12), malate is actively transported from the matrix to the cytosol, where malate dehydrogenase oxidizes L-malate to oxaloacetate and finally to pyruvate. The transporter (dicarboxylate carrier) is assumed to limit the rate of this pathway in a mechanism that follows Hill-type dynamics. The second regulation involves the enzyme pyruvate carboxylase, which replenishes oxaloacetate when needed. AcCoA is a positive allosteric modulator of this enzyme that follows Hill-type dynamics [58]. The regulations are modeled using the functions *U*_mal _= *U*^*a*^([oxal]; *V*_mal_, 1, ·*m*_*w*_) and *U*_oxal _= *U*^α ^([acc]; *V*_oxal_, 1, ·*m*_*w*_), for malate and oxaloacetate, respectively. As the concentration of AcCoA in the matrix exceeds the half saturation value, the flow of AcCoA into the cycle is enhanced by replenishing oxaloacetate.

The glycolysis process is modelled here as a part of the intermediate metabolism, and is described by the equations in Table 9.

**Table 9:** Glycolysis. State space equations for the glycolysis process, with the state variables *glu*, *g6p*, *pGt*, *g3p *are glucose, glucose-6-phosphate, 6-phosphogluconate and glyceraldehyde 3-phosp.

State space equation
[] = - *k*_*g*1 _[*glu*] + *u*_*g*, *feed*_
[] = *k*_*g*1 _[*glu*] - *k*_*g*2 _[*g*6*p*]·[NADP^+^] - *U*_*glyc*_*k*_*glyc *_[*g*6*p*]
[] = *k*_*g*2 _[*g*6*p*]·[NADP^+^] - *k*_*g*2 _[*pGt*]·[NADP^+^]
[] = *U*_*glyc*_*k*_*glyc *_[*g*6*p*] + *k*_*g*2 _[*pGt*]·[NADP^+^] - *k*_*g*2 _[*g*3*p*]·ATP

**Rate constants**

*k*_*g*1 _= 1/s
*k*_*g*2 _= 1/*μ*mol/s
*k*_*glyc *_= 1/*μ*mol/s

## Authors' contributions

The model and its differential derivatives was developed by NSB. Model analysis and manuscript were prepared by NR and NSB.
